# Hashimoto Encephalopathy in Pediatrics: Report of 3 Cases

**DOI:** 10.1016/j.aace.2020.11.008

**Published:** 2020-12-28

**Authors:** Anne-Marie D. Kaulfers, Samar K. Bhowmick

**Affiliations:** University of South Alabama, Department of Pediatric Endocrinology, Mobile, Alabama

**Keywords:** Hashimoto disease, pediatrics, hypothyroidism, psychology, CSF, cerebrospinal fluid, EEG, electroencephalogram, HE, Hashimoto encephalopathy, IV, intravenous, MRI, magnetic resonance imaging, NMDA, N-Methyl-D-aspartic acid, TPO, thyroid peroxidase, TSH, thyroid-stimulating hormone

## Abstract

**Objective:**

To describe the rare occurrence of pediatric Hashimoto encephalopathy in 3 patients.

**Methods:**

The patients, 9 to 13 years of age, presented with new-onset seizures and other neurologic symptoms, including hemiplegia, aphasia, and memory loss. Thyroid function tests and thyroid antibodies were measured. Magnetic resonance imaging (MRI) of the brain, cerebrospinal fluid analysis, and electroencephalography were also performed.

**Results:**

The first patient had a thyroid-stimulating hormone (TSH) level of 60 μIU/mL (range, 0.4-4.5), free T4 of 0.28 ng/dL (range, 0.7-1.6), and thyroid peroxidase antibody (TPO Ab) of 1243 IU/mL (range < 9). The MRI results indicated a hyperintense signal along the gyri and sulci with diffuse leptomeningeal enhancement bilaterally. The second patient had a TSH level of 25 μIU/mL, free T4 level of 0.7 ng/dL, and TPO Ab level of 3340 IU/mL. The MRI result was normal. The third patient, who was already on levothyroxine, had a TSH level of 17 μIU/mL, free T4 level of 0.81 ng/dL, and TPO Ab level of 1200 IU/mL. The MRI result was normal. All patients had significant elevation of protein in the cerebrospinal fluid and background slowing on electroencephalography. All patients were treated with high doses of intravenous methylprednisolone followed by oral prednisone and thyroid hormone replacement.

**Conclusion:**

These cases underscore the importance of thyroid function tests with antibodies in children presenting with acute neuropsychiatric manifestations, especially new-onset seizures without any identifiable cause. We believe that this condition is underdiagnosed in children, and a high index of suspicion is recommended.

## Introduction

The prevalence of Hashimoto thyroiditis in school-aged children is about 1.2%, and thyroid enlargement is noted in about 85% of children with positive thyroid antibodies.^1^ Although many children with high levels of thyroid antibodies remain asymptomatic, Hashimoto thyroiditis is the most common cause of hypothyroidism in children.^1^^,^^2^ Hashimoto encephalopathy (HE), a complication of Hashimoto thyroiditis, is rare in children. There are very few case reports on this condition, and most have been published in neurology journals. HE refers to a syndrome of persistent fluctuating neurologic and neuropsychologic deficits associated with elevated thyroid antibodies, specifically thyroid peroxidase (TPO) antibodies. We describe 3 pediatric patients who presented with various neuropsychological symptoms, cognitive impairment, and new-onset seizures. They were all treated at the University of South Alabama Children’s and Women’s Hospital within a 4-year time span. We believe that these 3 case reports will add to the limited knowledge of this condition in pediatrics.

## Case Reports

### Patient 1

A 9-year-old boy was admitted with new-onset seizures. He complained of severe headaches for weeks prior to the seizure and was diagnosed with hemiplegic migraines a year previously. On physical exam, he had right-sided hemiplegia, aphasia, and a moderate-sized goiter. Laboratory evaluation showed a normal complete blood count and normal metabolic profile. The cerebrospinal fluid (CSF) analysis was clear, with no pleocytosis, normal glucose, and a high protein level of 142 mg/dL (reference range, 15-45). An electroencephalogram (EEG) confirmed intermittent slowing over the right occipital region. Magnetic resonance imaging (MRI) results indicated a hyperintense signal along the gyri and sulci with diffuse leptomeningeal enhancement bilaterally. Thyroid function tests revealed a high thyroid-stimulating hormone (TSH) level, low free thyroxine level, and high levels of both thyroid antibodies (Table). His seizures were intractable with conventional antiepileptic medication, and he required intubation. He was diagnosed with HE and was started on 0.5 g intravenous (IV) methylprednisolone daily for 5 days, followed by oral prednisone 2 mg/kg/day. His seizures were controlled within 2 days along with resolution of aphasia and hemiplegia. He was discharged on a tapering dose of steroids, antiepileptic drugs, and thyroid hormone replacement.

**Table:** Thyroid Profile

Patient^a^	Patient sex/age	Free thyroxine^b^ (0.77-1.6 ng/dL)	Thyroid-stimulating hormone^b^ (0.4-4.5 μIU/mL)	Thyroid peroxidase antibody (<9 IU/mL)	Anti-thyroglobulin antibody (<4 IU/mL)^b^
1	Male/9 y	0.28	60.13	1243	12.9
2	Female/13 y	0.70	25	3340.5	354.9
3	Female/13 y	0.81	17.4	1200.4	19

a Thyroid-stimulating hormone and both thyroid antibody levels were very elevated in all 3 patients. Free thyroxine level was normal in patient 3 because she was already on levothyroxine.

b Normal range or value.

### Patient 2

A 13-year-old girl was admitted with generalized seizures. She was diagnosed with bipolar disorder and attention-deficit hyperactivity disorder a year previously. She did not have a goiter or any symptoms of hypothyroidism. Laboratory evaluation showed a normal complete blood count and normal metabolic profile. Her CSF was clear, with no pleocytosis, normal glucose level, and a high protein level of 135 mg/dL. An EEG showed intermittent diffuse bursts of slowing. MRI was normal. Thyroid function tests showed a high TSH level, low free thyroxine level, and high levels of both thyroid antibodies (Table). Conventional antiepileptic medication failed to control the seizures, and she also required intubation. She was diagnosed with HE and started on high-dose IV methylprednisolone followed by oral prednisone, the same doses as in our first patient. Her seizures were controlled after a few days, and she was discharged home on a tapering dose of prednisone, antiepileptic drugs, and thyroid hormone replacement.

### Patient 3

A 13-year-old girl was admitted with new-onset episodic memory loss, agitation, confusion, and depression, which then progressed to generalized tonic-clonic seizures. She was diagnosed with hypothyroidism by her pediatrician 2 months previously and was already on levothyroxine. Both her mother and older sister have hypothyroidism. She had a small goiter on physical exam. Laboratory evaluation showed a normal complete blood count and normal metabolic profile. The CSF was clear, with no pleocytosis, normal glucose level, and a high protein level of 83 mg/dL. EEG showed background slowing with intermittent spikes. An MRI showed a small left arachnoid cyst on the left side of the cerebellum but was otherwise normal. Her thyroid levels were repeated on this admission. She had a high TSH level, normal free thyroxine level, and high levels of both thyroid antibodies (Table). This patient was treated in the same manner as the other 2, with quick resolution of her seizures. She did not require intubation and was discharged home with thyroid hormone replacement, a tapering dose of prednisone, and antiepileptic drugs.

## Discussion

Since the first description of a case report by Brain et al in 1966,^3^ the adult incidence of HE has been reported to be 2.1 per 100 000. About one-fifth of the cases are presumed to be under 18 years of age with a female preponderance 4:1.^4^^,^^5^ Watemberg et al^2^ found 22 case reports of HE in the pediatric literature. Eighteen of those cases were those of girls between 8 and 17 years of age. Only 2 cases were diagnosed in patients under 10 years of age. HE is a syndrome of neurological symptoms with serological evidence of autoimmune thyroid disease. Thyroid dysfunction is variable and may range from hypothyroidism to a euthyroid state to occasionally hyperthyroidism.^5^^,^^6^ High levels of TPO antibodies are seen in 100% of cases, and high thyroglobulin antibody levels are seen in about 73% of cases.^5^, ^6^, ^7^ Antibody titers and severity of thyroid dysfunction do not correlate with the clinical presentation and severity of HE in most reports.^2^^,^^8^^,^^9^ In many cases, HE also coexists with other autoimmune diseases, such as lupus or Sjogren syndrome.^9^

The etiology of HE is incompletely understood, and the pathophysiology remains speculative. Autoimmune processes most likely play a major role, possibly autoimmune cerebral vasculitis. A recent thought also favors inflammatory responses to antineural antibodies.^10^^,^^11^ Other sources point to hormonal dysregulation as a possible etiology.^9^ Encephalitis caused by autoimmunity is now frequently recognized in the pediatric population, although the exact prevalence is unknown.^12^ One of the most frequently published causes of autoimmune encephalitis is an antibody against the N-Methyl-D-aspartic acid (NMDA) receptor, which was first described in 2007. Anti-NMDA encephalitis in children presents with behavior changes, seizures, and unusual movements involving the face and extremities. It is diagnosed by detecting anti-NMDA receptor autoantibodies in both the serum and CSF.^12^ There are several other known antibodies that cause encephalitis in the pediatric population, but most are not commercially available for testing.^12^ In our patients, the NMDA receptor and other autoantibodies were not tested because they were not easily accessible to our institution at that time. Because other antibodies were not measured and the prevalence of thyroid autoantibodies in the general population is 10% to 15%, there is a possibility of a false positive diagnosis of HE in our patients.^13^ The diagnostic criteria for HE, as proposed by Doherty,^10^ are reasonable. The criteria require that the patient have cognitive impairment with or without neuropsychologic symptoms. The patient might have seizures and stroke-like events with focal neurologic deficits. Elevated thyroid antibody levels, especially TPO, should be present in all cases to make the diagnosis, and the patient may have hypothyroid or euthyroid. CSF should have an elevated protein level but no pleocytosis, and the patient should respond well to corticosteroid therapy. The EEG abnormality could show mild-to-moderate generalized slowing, and MRI may be normal or show nonspecific changes.^10^ All of our patients fulfilled these criteria.

Although heterogeneous in presentation, 2 types of HE have been described, a vasculitis type and a diffuse type.^8^^,^^11^^,^^14^ The vasculitis type usually presents with repetitive stroke-like episodes, such has hemiparesis, aphasia, ataxia, and mild cognitive impairment. The diffuse–progressive type is associated with insidious onset of dementia, seizures, hallucinations, psychiatric episodes, or altered consciousness. The diffuse type appears to be more common, but neither presentation is exclusive, and significant overlap may occur. Furthermore, EEG, MRI, and CSF analyses cannot distinguish between the 2 subtypes.^8^^,^^14^ Our 3 patients represent the diffuse type.

Treatment of HE consists of high-dose methylprednisolone, 0.5-1 g IV daily for 3 to 5 days, followed by oral prednisone 2 mg/kg/day. Slow tapering of prednisone is advised as rapid tapering may bring about a relapse.^5^^,^^7^ If first-line treatment fails, IV immunoglobulin can be used, as well as other immunosuppressive drugs like cyclosporine, azathioprine, and/or methotrexate.^7^ However, those are rarely used in pediatrics.

Our first patient had a recurrence of seizures within 6 months, which may have been in response to the rapid reduction of prednisone. He was treated again with IV methylprednisolone followed by oral prednisone along with intravenous immunoglobulin monthly for 1 year. His antiepileptic medication was also adjusted. He is doing well now but with occasional hallucinations. Our second patient has had no seizures but continues to show intermittent hallucinations and depression. Our third patient was lost to follow up after 1 year.

## Conclusion

Both the etiology and pathophysiology of HE should be further elucidated. Despite speculative origins, HE remains a treatable encephalopathy in children. Physicians must be aware that younger patients may present with a diverse clinical picture, including new-onset seizures or various neuropsychiatric symptoms. These 3 cases underscore the importance of thyroid function tests and thyroid antibody levels for any child who presents with these symptoms, especially new-onset seizures without any identifiable causes. Although recurrence may occur, HE is readily treatable, steroid-responsive, and carries a good prognosis as opposed to other encephalopathies in children. We believe that this condition is under diagnosed in the pediatric age group, so a high index of suspicion is advised.

## Disclosure

The authors have no multiplicity of interest to disclose.
